# Utilizing artificial intelligence for the detection of hemarthrosis in hemophilia using point-of-care ultrasonography

**DOI:** 10.1016/j.rpth.2024.102602

**Published:** 2024-10-23

**Authors:** Pascal N. Tyrrell, María Teresa Alvarez-Román, Nihal Bakeer, Brigitte Brand-Staufer, Victor Jiménez-Yuste, Susan Kras, Carlo Martinoli, Mauro Mendez, Azusa Nagao, Margareth Ozelo, Janaina B.S. Ricciardi, Marek Zak, Johannes Roth

**Affiliations:** 1 Department of Medical Imaging, University of Toronto, Toronto, Ontario, Canada; 2 Institute of Medical Science, University of Toronto, Toronto, Canada; 3 Department of Statistical Sciences, University of Toronto, Toronto, Canada; 4 Hematology Department, Hospital Universitario La Paz-IdiPaz, Autónoma University, Madrid, Spain; 5 Indiana Hemophilia & Thrombosis Center, Indianapolis, Indiana, USA; 6 Children’s Hospital of Philadelphia, Pennsylvania, USA; 7 Novo Nordisk Healthcare, Zürich Switzerland; 8 Mohawk College, Institute for Applied Health Sciences, McMaster University, Hamilton, Ontario, Canada; 9 Department of Health Sciences, University of Genoa, Genova, Italy; 10 IRCCS Ospedale Policlinico San Martino, Genova, Italy; 11 Department of Blood Coagulation, Ogikubo Hospital, Tokyo, Japan; 12 Hemocentro UNICAMP, University of Campinas, Campinas, Brazil; 13 Novo Nordisk A/S, Søborg, Denmark; 14 Children’s Hospital of Central Switzerland, Luzern, Switzerland

**Keywords:** artificial intelligence, hemarthrosis, hemophilia, joint, ultrasonography

## Abstract

**Background:**

Recurrent hemarthrosis and resultant hemophilic arthropathy are significant causes of morbidity in persons with hemophilia, despite the marked evolution of hemophilia care. Prevention, timely diagnosis, and treatment of bleeding episodes are key. However, a physical examination or a patient’s assessment of musculoskeletal pain may not accurately identify a joint bleed. This difficulty is compounded as hemophilic arthropathy progresses.

**Objectives:**

Our system aims to utilize artificial intelligence and ultrasonography (US; point-of-care and handheld) to enable providers, and ultimately patients, to detect joint bleeds at the bedside and at home. We aimed to develop and assess the reliability of artificial intelligence algorithms in detecting and segmenting synovial recess distension (SRD; an indicator of disease activity) on US images of adult and pediatric knee, elbow, and ankle joints.

**Methods:**

A total of 12,145 joint exams, comprising 61,501 US images from 7 international healthcare centers, were collected. The dataset included healthy participants and adult and pediatric persons with hemophilia, with and without SRD. Images were manually labeled by 2 experts and used to train binary convolutional neural network classifiers and segmentation models. Metrics to evaluate performance included accuracy, sensitivity, specificity, and area under the curve.

**Results:**

The algorithms exhibited high performance across all joints and all cohorts. Specifically, the knee model showed an accuracy of 97%, sensitivity of 96%, specificity of 97%, and an area under the curve of 0.97 in SRD. High Dice coefficients (80%-85%) were achieved in segmentation tasks across all joints.

**Conclusion:**

This technology could assist with the early detection and management of hemarthrosis in hemophilia.

## Essentials

• Despite advances in care, joint bleeds are a major cause of morbidity in persons with hemophilia.
• Artificial intelligence plus ultrasonography may enhance early detection of joint bleeds.
• This project aimed to test the reliability of artificial intelligence in detecting joint bleeds from images.
• The artificial intelligence algorithm showed a high level of performance in detecting joint bleeds.

## 1 Introduction

Hemophilia care has transformed in the past decade, but joint bleeds continue to occur in persons with hemophilia. Hemophilic arthropathy, characterized by synovial proliferation and osteochondral damage from recurrent bleeding episodes and inflammation, diminishes quality of life and remains a significant cause of morbidity in this patient population [[1], [2], [3], [4]].

Prevention, early detection, and management of hemarthrosis in persons with hemophilia are essential for the preservation of joint health. The current evaluation and management approach of painful and acute musculoskeletal episodes in persons with hemophilia is empirical and subjective at best, and significant discrepancies continue to exist between patient/physician-perceived etiology of joint pain and ultrasound findings [[5], [6], [7]]. This results in the over- and undertreatment of joint bleeding episodes with hemostatic factor therapy and underutilization of physical therapy, anti-inflammatory agents, and other important treatment modalities. Lastly, a gap continues to exist in our ability to detect early or subclinical hemophilic arthropathy and/or temporally follow it over time.

Ultrasonography (US) is a rapid, convenient, safe, cost-effective, and readily accessible imaging modality with an ability to detect joint effusions and soft tissue changes that is comparable with the gold standard, magnetic resonance imaging [8,9]. In addition, US does not require sedation in children. With proper training and increasing distribution of low-cost US devices and probes that can be connected to handheld devices to deliver high-quality images, US has emerged as a promising tool in the modern care of persons with hemophilia for the evaluation of joint bleeding episodes, musculoskeletal injuries, and monitoring of hemophilic arthropathy over time. Access to rapid, accurate, and expert image analysis and interpretation remains suboptimal and is of concern [10]. In pediatric patients, analyzing the immature skeleton, growth plates, and secondary ossification centers requires advanced knowledge, presenting challenges for even skilled sonographers and sonologists [11].

Artificial intelligence (AI)/machine learning (ML) may be able to support the interpretation of US images of joints in persons with hemophilia. The exponential growth of computer power and available, affordable storage have opened avenues for research and innovation and provided the opportunity to address problems that were previously difficult to overcome [12]. The feasibility of using AI/ML to detect joint bleeds has been demonstrated [13,14], and the application of AI/ML to pattern recognition in medical images has been successful in enhancing clinical decision-support software solutions, especially in situations that require standardization and processing of complex problems [15,16].

Synovial recess distension (SRD) can occur due to several reasons, including the presence of blood, synovial fluid, or synovial hypertrophy, so SRD may be used as an indirect surrogate for disease activity in a joint but is not specific for joint bleeds [17]. This surrogate may be particularly helpful in assessing joint bleeds in persons with hemophilia, especially in the context of a suspected bleed due to the presence of symptoms that are commonly associated with bleeding (such as “tingling sensation,” pain, swelling, or loss of range of motion) [18]. It may also be useful for screening purposes in asymptomatic persons with hemophilia. The utility of using SRD as a surrogate is also emphasized by the challenge of using US to reliably detect blood itself during a joint bleed, especially in the subacute phase. The detection of an SRD on US would therefore be a first but important step, followed by further assessment of differential diagnosis.

Here, we develop and test a convolutional neural network (CNN) deep learning algorithm for US scans of the knee, elbow, and ankle joints to detect SRD in healthy participants and adult and pediatric persons with hemophilia. We test the reliability of this algorithm in the detection of pathologic SRD in healthy children and adults, as well as persons with hemophilia of all ages.

## 2 Methods

### 2.1 Data collection and patient population

US images were procured from 4 distinct patient populations for both adult and pediatric participants: healthy participants with no SRD (healthy controls), healthy participants with a normal amount of physiological fluid but with incidental findings of SRD (healthy cases), persons with hemophilia with no hemarthrosis (hemophilia controls), and persons with hemophilia with a high clinical suspicion of hemarthrosis (hemophilia cases). Therefore, this paper will refer to the following 4 cohorts: healthy adult participants, adult persons with hemophilia, healthy pediatric participants, and pediatric persons with hemophilia.

**Figure 1:**
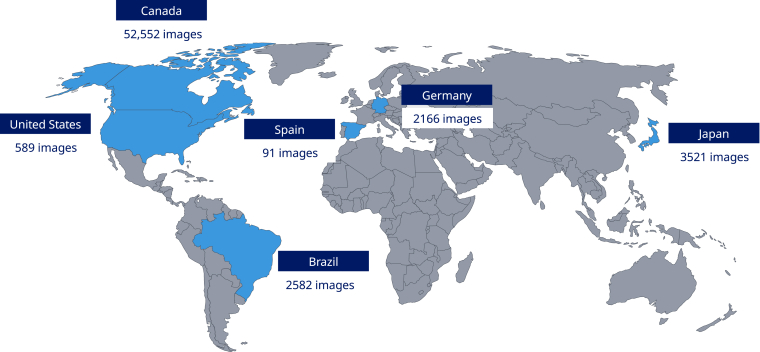
Image source healthcare center locations.

It should be noted that participants presenting with both acute and subacute hemarthrosis were included, and that a small amount of intra-articular fluid is normal in patients and healthy participants who are asymptomatic. Exam images were obtained from 7 healthcare centers across the world (Figure 1). Each center obtained ethics approval for this study (Radiology Information System [RIS] protocol #39361), and consent (either opt-out or informed) was obtained from all participants prior to study inclusion [19]. Exams were completed with portable, nonhandheld point-of-care US devices (LogiqE R7, General Electric; FUJIFILM Sonosite, Inc). The overall dataset comprised a total of 10,439 adult joint exams (56,276 US images) and 1706 pediatric joint exams (5225 US images). After quality assessment, a total of 9842 exams (from 52,350 US images) for the knee, 886 exams (from 3120 US images) for the elbow, and 1417 exams (from 6031 US images) for the ankle were used (Table 1). Images were captured according to the Hemophilia Early Arthropathy Detection with Ultrasound (HEAD-US) protocol and were intended to demonstrate midsagittal, longitudinal images of the olecranon recess of the elbow, suprapatellar recess of the knee, and anterior tibiotalar recess of the ankle [20].

**Table 1:** Comprehensive overview of collected ultrasonography imaging data.

Patient population		No. of exams (no. of images)		
		Knee	Elbow	Ankle
**Healthy controls**	**Adult**	4092 (23,667)	93 (544)	259 (1350)
	**Pediatric**	151 (588)	84 (195)	73 (382)
**Healthy cases**	**Adult**	4162 (23,666)	93 (543)	395 (2099)
	**Pediatric**	181 (524)	79 (187)	133 (290)
**Hemophilia controls**	**Adult**	329 (1177)	179 (518)	149 (506)
	**Pediatric**	293 (770)	83 (227)	134 (456)
**Hemophilia cases**	**Adult**	284 (1185)	203 (517)	201 (504)
	**Pediatric**	350 (773)	72 (389)	73 (444)

### 2.2 Image labeling and quality assessment

All US images were labeled by extraction from the radiologic report for negative cases (no SRD) when available or via assessment by 2 experts in sonography with extensive clinical experience in musculoskeletal US. The experts were asked to evaluate the presence of SRD and delineate the contour of the SRD in the US images using the labeling tool Label Studio (HumanSignal). The experts were blinded to the patients’ clinical information to minimize potential bias.

The manually labeled images were then used in a high-quality dataset for training and validating the AI algorithms. The case:control ratio for all 4 cohorts was balanced based on the presence of SRD. The test set (used to evaluate the performance of the algorithm; 20% of samples) was randomly selected separately for each data cohort to ensure the representation of all patient populations in the evaluation of the AI algorithms. All images in a study were assigned to either training or a test set to avoid data leakage.

### 2.3 Algorithm development for SRD detection

#### 2.3.1 Knee base model

A binary CNN (BCNN) classifier was developed to detect knee SRD in both adult and pediatric patients. Because empirical experiments showed a similar model performance on adult and pediatric exams, both data populations were included to enrich the training dataset. The training process involved data augmentation techniques such as rotation, scaling, and flipping to increase the diversity of the training set and improve the model’s generalization capability. The model’s hyperparameters were tuned using cross-validation, and the final configuration was selected based on its performance on a validation set. The model architecture was based on EfficientNet-B4 (Google Brain) and was trained for a total of 50 epochs, with a learning rate of 0.001 (with a decreasing scheduler), binary cross-entropy loss, and the Adam optimizer [21]. A batch size of 64 images was selected due to the size of the dataset. The model training was initialized from a pretrained state using the ImageNet dataset (Stanford Vision Lab) [22]. Two GeForce RTX 2080 Ti graphics processing units (Nvidia) were used for model training.

#### 2.3.2 Elbow and ankle models

A BCNN classifier was independently developed to detect SRD in the elbow and ankle joints of both adult and pediatric patients. Pretraining on knee exams allowed the BCNN to learn US characteristics (ie, noise, tissue texture, and pixel intensity) and subsequently facilitated generalizable training on the elbow and ankle US datasets despite the disparity in cases of elbow and ankle SRD compared with cases of knee SRD. Similar data augmentation techniques, hyperparameter tuning strategies, and training environments were used to optimize the model’s performance. However, batch size and optimizer updates were reduced to accommodate the difference in dataset size.

### 2.4 Algorithm development for SRD segmentation

In addition to the BCNN classifier, segmentation models were constructed for knee SRD, as well as for the elbow and ankle joints, using a DeepLab V3 (Google) architecture with a ResNet101 encoder (Microsoft Research Asia) [23]. This deep learning approach was chosen for its ability to efficiently learn complex patterns in medical images, enabling the precise segmentation of SRD. The models were trained similarly to the BCNN classifiers using the knee cohort as the starting point for the elbow and ankle. Images from adult and pediatric patients were combined into a single training dataset per joint. A total of 150 epochs were used in the training with binary cross-entropy loss, a learning rate of 0.0001, and the Adam optimizer.

### 2.5 Performance evaluation

The AI algorithms were evaluated on an independent test set containing images from all data populations, which was not involved in model training or optimization. The performance of the BCNN classifiers and segmentation models was evaluated using standard metrics such as accuracy, sensitivity, specificity, area under the receiver operating characteristic curve (area under the curve [AUC]), and the Dice coefficient for segmentation models. The Dice coefficient is a widely used measure for the evaluation of medical image segmentation algorithms [[24], [25], [26]]. By providing numerical values, it allows for a comparison of different segmentation results and an assessment of the quality of the segmentation. In addition, we included the results of the 5-fold cross-validation setting used to train and validate each model.

## 3 Results

The AI-based classifiers and segmentation models exhibited high accuracy in identifying and delineating SRD in both adult and pediatric populations with and without hemophilia across knee, elbow, and ankle joints. The high performance of the proposed methodology was substantiated by an AUC exceeding 0.9 for all 3 joints.

### 3.1 Detection of SRD

#### 3.1.1 Knee base model

The BCNN classifier was effective in detecting the presence of SRD in knee joints, both in adult and pediatric patients, with or without hemophilia. The models demonstrated robustness, showing no signs of overfitting, and achieved convergence approximately at the 40th epoch. The knee joint model served as the foundational architecture for subsequent models targeting the elbow and ankle joints, thereby enhancing initial training and resulting in improved detection metrics. The knee model outperformed the models for the elbow and ankle joints, a result primarily attributed to the larger sample size of knee exams in the dataset. With an accuracy of 97%, a sensitivity of 96%, a specificity of 97%, and an AUC of 0.97, the model exhibited high reliability in distinguishing between case and control images in persons with and without hemophilia. Although the model’s performance showed a marginal decline when tested on separate pediatric patient groups, it still maintained all key metrics above the 90% threshold. Comprehensive results are provided in Table 2.

**Table 2:** Performance evaluation of synovial recess distension detection and segmentation models in the knee joint.

Knee (AUC = 0.97)	Test set				K-fold, % mean ± SD (k = 5)			
	Accuracy, %	Sensitivity, %	Specificity, %	Dice, %	Accuracy, %	Sensitivity, %	Specificity, %	Dice, %
**Overall performance**	97	96	97	85	95 ± .2	95 ± .5	96 ± .5	85 ± .6
**Healthy adult participants**	98	98	98	83	96 ± .1	97 ± .5	96 ± .5	84 ± .0
**Adult persons with hemophilia**	95	89	98	91	93 ± 1.2	87 ± 1.0	94 ± 1.9	90 ± .5
**Healthy pediatric participants**	90	84	96	89	89 ± 1.3	87 ± 3.0	87 ± 2.3	90 ± .5
**Pediatric persons with hemophilia**	91	95	89	88	89 ± 2.8	86 ± 3.9	93 ± 3.1	87 ± 1.7

AUC, area under the curve; SD, standard deviation.

#### 3.1.2 Elbow and ankle models

When the BCNN classifiers were retrained separately using US datasets for the elbow and ankle joints, we observed a significant improvement in performance metrics. Specifically, models pretrained on knee US data demonstrated a 4% increase in accuracy compared with those without such pretraining. Comparative evaluations on test sets revealed that the model trained with ankle US data surpassed the performance of the model trained on elbow joint data.

For the elbow dataset (pretrained on knee US data), test metrics indicated an accuracy of 87%, a sensitivity of 92%, a specificity of 86%, and an AUC of 0.92. In contrast, the ankle dataset (also pretrained on knee US data) yielded markedly superior metrics, with an accuracy of 94%, a sensitivity of 92%, a specificity of 97%, and an AUC of 0.93. Upon analyzing the model’s performance across 4 distinct patient cohorts, we found that all groups exhibited comparable performance except for the healthy pediatric cohort, which showed a minor decline in detection efficacy. Comprehensive results are presented in Tables 3 and 4.

**Table 3:** Performance evaluation of synovial recess distension detection and segmentation models in the elbow joint.

Elbow (AUC = 0.92)	Test set				K-fold (k = 5)			
	Accuracy, %	Sensitivity, %	Specificity, %	Dice, %	Accuracy, %	Sensitivity, %	Specificity, %	Dice, %
**Overall performance**	87	92	86	82	88 ± 1.0	85 ± 3.1	91 ± 3.1	83 ± 1
**Healthy adult participants**	92	95	88	67	96 ± 1.7	96 ± 3.3	96 ± 2.9	76 ± 2.1
**Adult persons with hemophilia**	88	91	84	91	87 ± 1.4	82 ± 3.9	92 ± 1.2	89 ± .5
**Healthy pediatric participants**	85	89	82	90	85 ± 2.6	87 ± 1.4	82 ± 2.3	81 ± 3.9
**Pediatric persons with hemophilia**	89	90	86	96	86 ± 3.3	84 ± 2.6	88 ± 3.1	92 ± .7

AUC, area under the curve; SD, standard deviation.

**Table 4:** Performance evaluation of synovial recess distension detection and segmentation models in the ankle joint.

Ankle (AUC = 0.93)	Test set				K-fold, % mean ± SD (k = 5)			
	Accuracy, %	Sensitivity, %	Specificity, %	Dice, %	Accuracy, %	Sensitivity, %	Specificity, %	Dice, %
**Overall performance**	94	92	97	80	91 ± 1.2	91 ± 1.2	90 ± 1.9	81 ± 1.2
**Healthy adult participants**	97	97	99	77	93 ± 1.7	95 ± 1.3	91 ± 2.6	80 ± 1.4
**Adult persons with hemophilia**	93	90	97	80	89 ± 2.4	87 ± 5.1	92 ± 1.6	83 ± 3.0
**Healthy pediatric participants**	87	85	90	77	86 ± 1.8	85 ± 3.1	87 ± 2.5	71 ± 4.7
**Pediatric persons with hemophilia**	94	88	97	89	92 ± 3.0	91 ± 2.6	93 ± 1.7	94 ± .0

AUC, area under the curve; SD, standard deviation.

### 3.2 Segmentation of SRD in adult and pediatric patients

**Figure 2:**
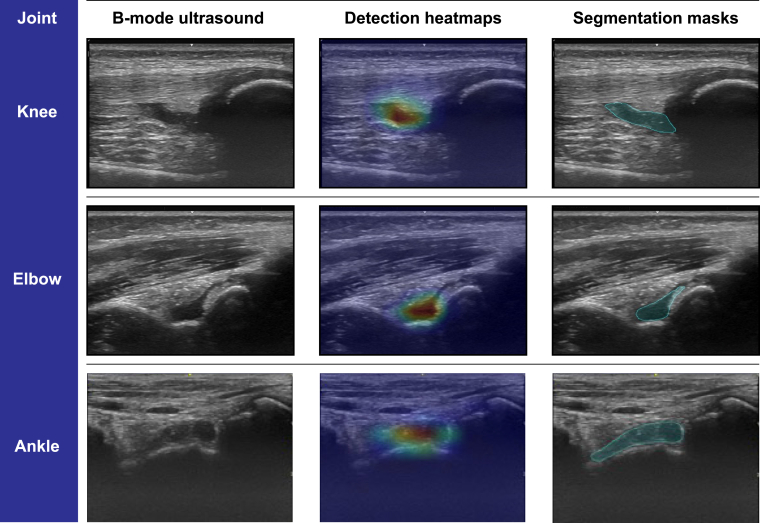
Visualization of prediction results on synovial recess distension ultrasonography images of the knee, elbow, and ankle. This figure displays, from left to right, gray-scale ultrasonography images, their respective detection heatmaps from the binary complex neural network (red zones indicate areas of greater focus), and the segmentation masks produced by the segmentation model. A detection heatmap (Grad-CAM) in a binary complex neural network highlights the regions of an image that heavily influence the network’s decision between 2 classes, providing a visual indication of important features used for classification. The visualization provides a clear demonstration of the model’s ability to accurately detect and delineate synovial recess distension across various joint types and patient populations.

We designed and evaluated segmentation models optimized for the identification of SRD in the knee, elbow, and ankle joints, incorporating both adult and pediatric radiographic data sets. Notably, the model-generated annotations exhibited more rounded contours of the SRD. This contrasts with the often angular and simplified outlines produced by expert annotators—a discrepancy largely attributed to time constraints during acquisition and the inherent limitations of existing annotation tools. This observation was corroborated by the segmentation metric used to assess the model’s performance. Successful segmentation of SRD was achieved across all 3 joints, as evidenced by high Dice coefficients of 85%, 82%, and 80% for the knee, elbow, and ankle joints, respectively. Interestingly, upon analyzing performance across 4 patient cohorts, we found that the model was most effective in pediatric persons with hemophilia and least effective in healthy adult participants. Representative samples illustrating the model’s segmentation capabilities are presented in Figure 2.

## 4 Discussion

Currently, joint bleeding episodes in persons with hemophilia are diagnosed and treated empirically, and while recommended, objective imaging evaluation is rarely used. The utility of US, including point-of-care ultrasonagraphy (POCUS) in the evaluation and management of hemarthrosis, has been demonstrated, and reliance on only patient-reported symptoms and clinical examination may result in over- and undertreatment with hemostatic factor therapy [6,7]. Currently, and as factor and nonfactor joint prophylaxis therapies improve, some joint bleeding episodes may go undetected due to a lack of overt symptoms (thereby deemed a subclinical bleed) and may result in joint damage, especially if repeated and untreated [27,28]. Regular screening and routine surveillance of joint health over time may detect such bleeds and signs of early hemophilic arthropathy, allowing for early intervention at a stage when the pathology may be treatable and/or preventable [29]. Lastly, in the context of clinical trials of newer hemostatic therapies, POCUS is used to document joint health [30] and could potentially be used to objectively measure joint bleeds.

Despite the evidence supporting the use of POCUS in persons with hemophilia, the adoption of this technology has been limited by the difficulty in obtaining rapid, accurate, and expert interpretation and analysis of the acquired images. Persons with hemophilia are generally advised to manage joint bleeds at home using subjective judgment. They typically visit the hemophilia treatment center if symptoms continue despite initial treatment or when patients, such as infants, cannot effectively communicate their symptoms. This study demonstrates the feasibility of combining US and AI to create a system that can assist with the detection of joint bleeds by expediting the imaging of symptomatic and asymptomatic joints in persons with hemophilia. This may be used to complement the current approach by providing more conclusive evidence of joint bleeds for practitioners.

This study was focused on developing and validating a CNN-based deep learning algorithm for the detection and confirmation of joint bleeds in both adults and children with hemophilia using US. The dataset included US images of the knee, elbow, and ankle joints collected from 7 healthcare centers worldwide. The algorithm showed high accuracy in identifying pathologic SRD across the elbows, knees, and ankles across the patient populations. Specifically, the knee model exhibited an accuracy of 97% and an AUC of 0.97, setting a strong foundation for subsequent elbow and ankle models. Pretraining on knee US data improved the accuracy of the elbow and ankle models by 4%. Moreover, the study introduced segmentation models that achieved high Dice coefficients, particularly excelling in the cohorts of pediatric participants with hemophilia. The results demonstrate the potential of AI in offering fast, reliable, and potentially cost-effective solutions for the detection and management of joint bleeds in persons with hemophilia.

This study provides compelling evidence for the efficacy of AI-based models in identifying and segmenting SRD on US images. The approach of using SRD as a surrogate for the detection of joint bleeds may prove to be reliable and practically efficient, as the identification of blood can be challenging despite existing US criteria for blood sonographic appearance. As touched on previously, the challenge is in part due to the overlap of sonographic characteristics between blood and synovial fluid, as well as synovial hypertrophy. Identifying blood is particularly challenging in subacute joint bleeds with the changing morphology of an organizing blood clot and in the presence of hemophilic arthropathy. Serial US imaging of SRD and evaluation of changes in recess size, recess content, echogenicity, and sonographic appearance may allow for the identification of new and/or acute bleeds in persons with hemophilia who have some abnormalities and/or joint disease at baseline.

### 4.1 Study limitations

Despite the encouraging results of this study, some limitations remain. First, the dataset includes images from multiple international healthcare centers, but demographic information was removed prior to data transfer. This limitation hindered our ability to assess the efficacy of the proposed algorithms across diverse populations. Second, the study depends on expert decisions made from still B-mode images without referencing the US cine-loop. This approach may be limited to the expert for clinical assessment, resulting in the potential for human error or bias. Third, we observed a decline in model performance when applied to pediatric datasets, possibly due to their lower representation in the overall data pool but also due to the significant same joint image variation across different age groups as the skeleton matures. For example, the epiphyseal cartilage is anechoic and, in the absence of an interface sign secondary to acoustic impedance, is difficult to distinguish from a simple effusion. Similarly, it is also difficult to interpret US images of the older population due to issues such as joint deformation and bone structures that are harder to identify. Thus, further research is needed to train and fine-tune the algorithms for all stages of development in the pediatric demographic and across different age groups. It should also be acknowledged that these data do not account for the heterogeneity of US technologies and operators. For example, the algorithms will need to be validated on handheld US images because although the image resolution of handheld probes is improving, it is still typically less compared with POCUS (laptop and/or cart-based devices), which could affect accuracy. All the limitations mentioned indicate avenues for future research and should be considered when interpreting the study’s findings.

## 5 Conclusions

There remains a considerable unmet need in hemophilia regarding the rapid and accurate detection of joint bleeds. As an extension to current hemophilia care and providers’ clinical assessments, a device that provides patients and providers with the means to diagnose joint bleeds rapidly and accurately could significantly increase patient quality of life, prevent unnecessary treatment, and ensure that bleeds do not go undetected. This device could also prove valuable in clinical trials by providing an objective means of measuring joint bleeds. This study establishes the feasibility of such a device, with the developed AI algorithms demonstrating high performance in detecting and segmenting SRD in US images. To provide AI-assisted interpretation of images, the images must be acquired first. As handheld devices become more widely available, an interesting avenue for further research may be to explore the use of computer-assisted algorithms to guide probe placement in more inexperienced users, including patients.
